# Fatal pulmonary embolism after surgery for small intestinal necrosis caused by idiopathic portal vein thrombosis: a case report

**DOI:** 10.1186/s40792-024-01900-2

**Published:** 2024-04-18

**Authors:** Hitoshi Iwasaki, Hideo Uehara, Yugo Fujimoto, Hirofumi Hasuda, Eiji Kusumoto, Yuichi Hisamatsu, Rintaro Yoshida, Yoshihisa Sakaguchi, Tetsuya Kusumoto

**Affiliations:** 1 Department of Gastrointestinal Surgery and Clinical Research Institute Cancer Research Division, NHO Kyushu Medical Center, 1-8-1 Jigyohama, Chuo-Ku, Fukuoka, 810-8563 Japan; 2 Department of Cardiology, NHO Kyushu Medical Center, 1-8-1 Jigyohama, Chuo-Ku, Fukuoka, 810-8563 Japan

**Keywords:** Portal vein thrombosis, Pulmonary embolism, Venous thromboembolism

## Abstract

**Background:**

Portal vein thrombosis (PVT) and venous thromboembolism (VTE) both result from partial or complete occlusion of a blood vessel by a blood clot. The prognosis of PVT is generally good; however, PVT with VTE, including pulmonary embolism (PE), has a high mortality rate. We report here a case of PE after surgery for small intestinal necrosis caused by idiopathic PVT.

**Case presentation:**

A 69-year-old female attended our hospital with a chief complaint of upper abdominal discomfort, and was diagnosed with necrosis of the small intestine as a result of unexplained PVT. She underwent partial resection of the small intestine. On the second postoperative day, she suffered from respiratory distress and went into cardiopulmonary arrest. The patient recovered following cardiopulmonary resuscitation, but PE was detected. Extracorporeal veno-arterial cardiopulmonary resuscitation and anticoagulation therapy were initiated immediately and the thrombus was aspirated as much as possible. Two days later, extracorporeal veno-arterial cardiopulmonary resuscitation was withdrawn and anticoagulation therapy was continued. The patient subsequently recovered with no neurological damage and was discharged on day 26 after the above procedure.

**Conclusions:**

Idiopathic PVT is often associated with VTE, and a prompt diagnosis and intervention may result in a good prognosis.

## Background

Portal vein thrombosis (PVT) generally has a good prognosis [1]; however, PVT is occasionally complicated with venous thromboembolism (VTE), including pulmonary embolism (PE), which has a high mortality rate [2, 3]. Although VTE is frequently fatal, appropriate intervention is known to improve its outcome [4, 5]. Herein, we report the case of a patient who developed postoperative PE resulting in cardiopulmonary arrest following surgery for intestinal necrosis caused by idiopathic PVT.

## Case presentation

**Fig. 1:**
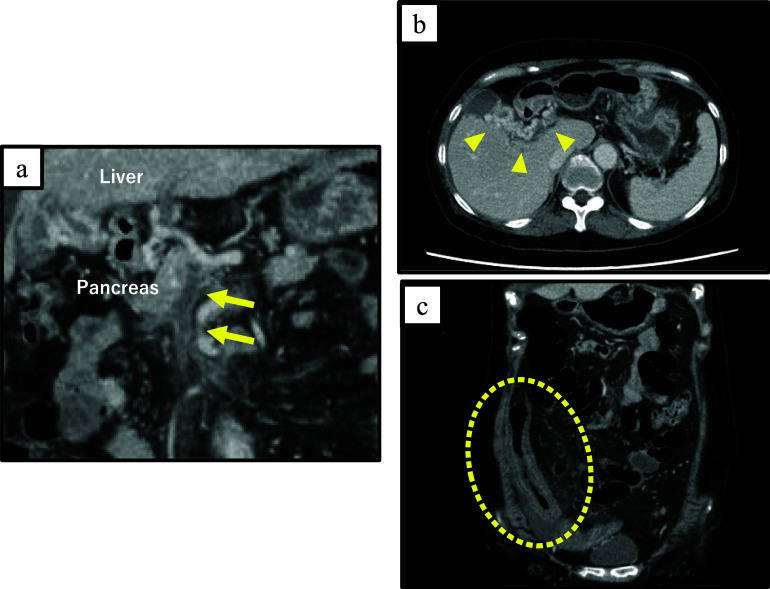
Contrast-enhanced computed tomography. **a** Portal vein thrombosis and mesenteric vein thrombosis from the superior mesenteric vein (arrow). **b** Cavernous transformation at the porta hepatis (arrowheads). **c** Poor contrast area of the small bowel loops (dotted line)

**Fig. 2:**
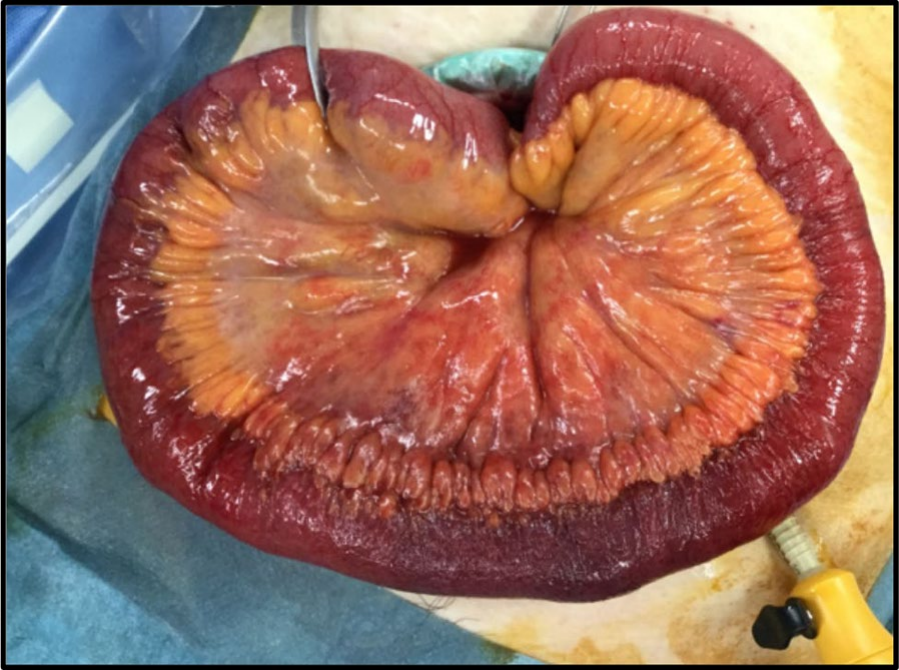
The terminal 30 cm of the ileum was discolored reddish black indicating intestinal necrosis

**Fig. 3:**
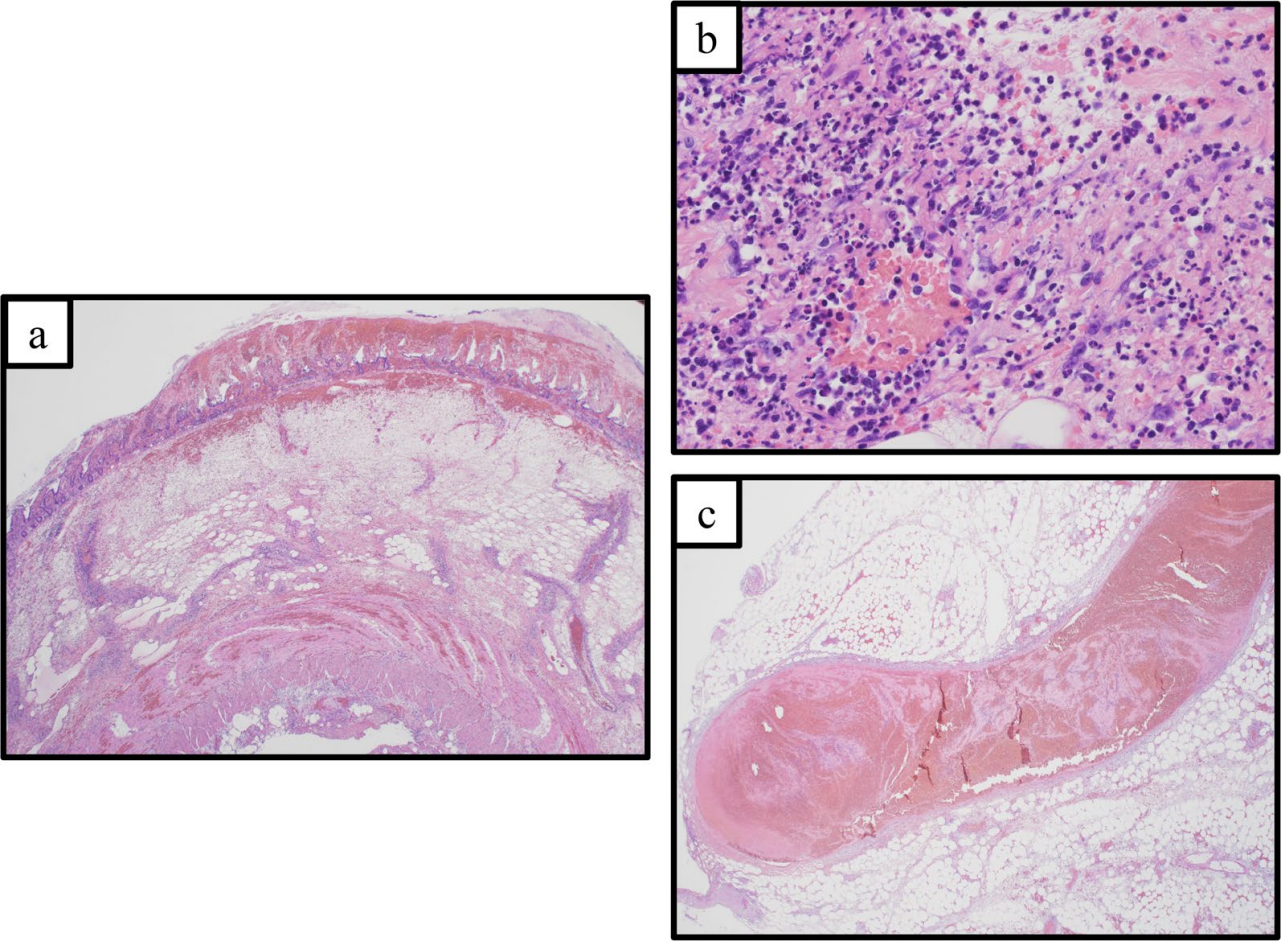
Pathological image of resection specimen. **a** Pathology specimen showing segmental whole circumferential mucosal necrosis with congested and edematous mucosa. **b** Section showing widely spreading mucosal necrosis, severe submucosal edema, and congestion, accompanied by transmural chronic active inflammatory infiltrate. **c** Large hematomas or thrombi we also observed in the peritoneal large veins. (Hematoxylin and eosin staining; **a** × 3; **b** × 40; **c** × 15.)

**Fig. 4:**
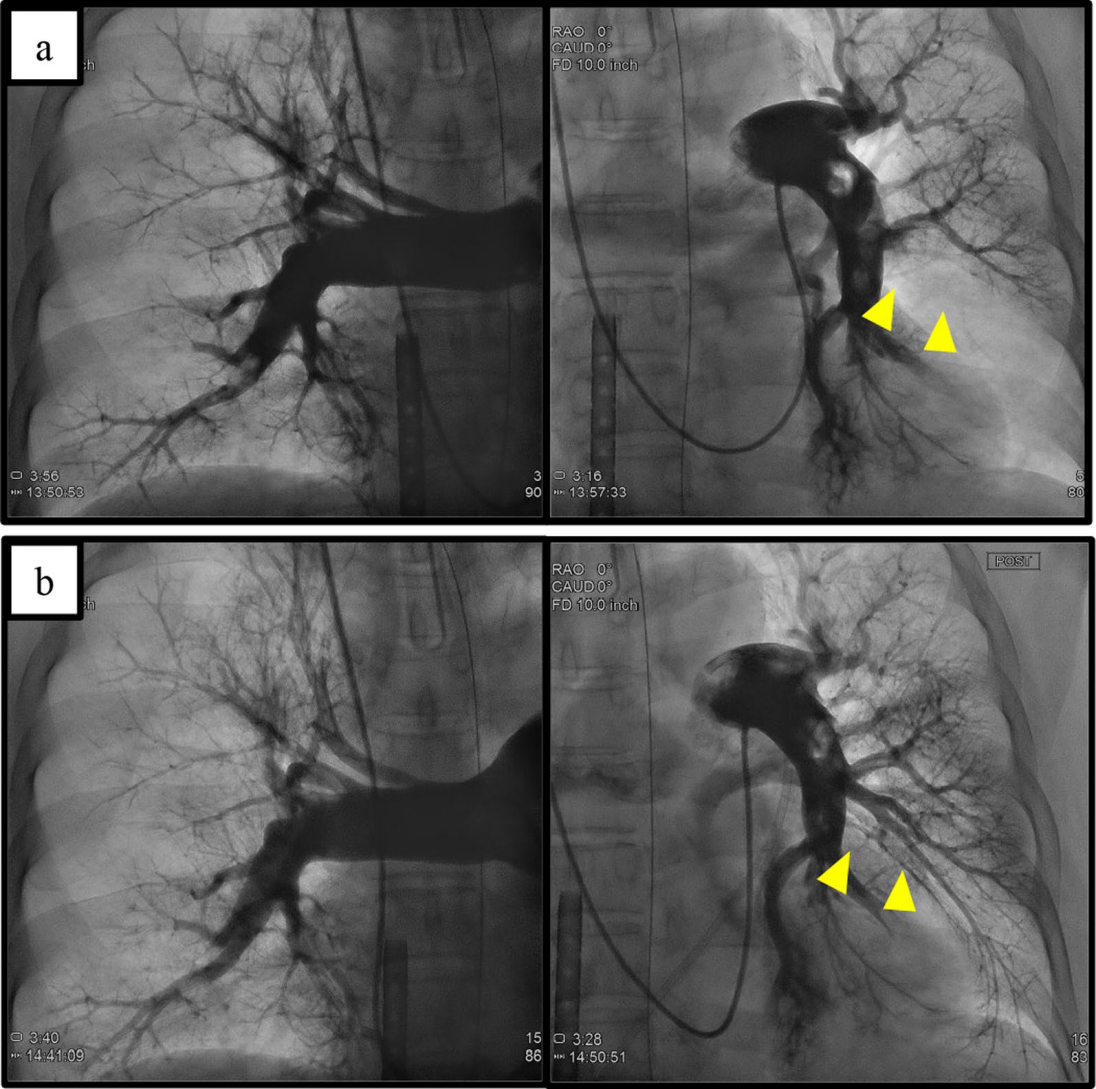
Angiography of pulmonary arteries. **a** Thrombus translucency was observed in the peripheral aspect of bilateral pulmonary arteries. There was a significant thrombus in the left > right pulmonary artery (arrowheads). **b** Thrombus aspiration was performed three times in the left pulmonary artery and once in the right pulmonary artery to remove the dark red thrombus. Some of the thrombus translucency disappeared (arrowheads)

**Fig. 5:**
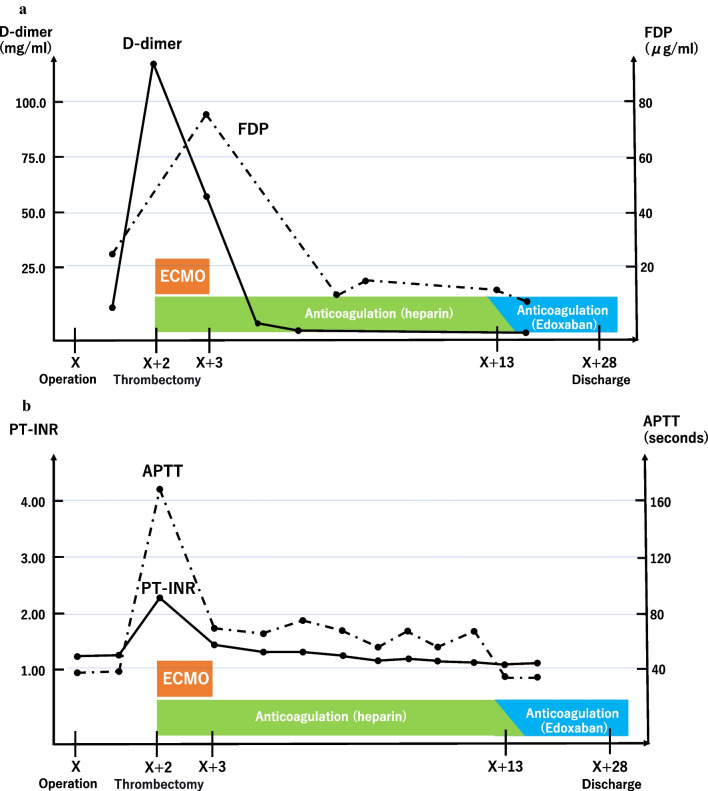
Summary of post-hospitalization progress. **a** Rapid increase in D-dimer and fibrin degradation products (FDP) from the postoperative period to the day of pulmonary embolism (PE) onset. **b** After the onset of PE, management was continued with heparin using the activated partial thromboplastin time (APTT) as an indicator. The patient was also changed to a direct oral anticoagulant 13 days after the initial surgery. ECMO: extracorporeal membrane oxygenation, PT-INR: prothrombin time-international normalized ratio

A 69-year-old female patient with no specific past medical history, including liver disease, attended our hospital with a complaint of upper abdominal discomfort for the past 7 days. Physical examination showed mild abdominal distension and tenderness in the right lower abdomen. Blood count and biochemistry results showed an elevated total leukocyte count (20,500/cm^3^) and elevated C-reactive protein (12.28 mg/dL). Contrast-enhanced computed tomography of the abdomen showed PVT and mesenteric vein thrombosis (MVT) and cavernous transformation at the porta hepatis, with poor contrast area of the small bowel loops (Fig. 1). She had no risk factors for PVT, leading to a diagnosis of idiopathic PVT. She was diagnosed with intestinal necrosis due to PVT and underwent emergency surgery. The terminal 30 cm of the ileum was discolored reddish black, indicating intestinal necrosis (Fig. 2). The necrotic area was resected and reconstructed with a functional end-to-end anastomosis. Pathological examination of the resected specimen revealed segmental whole circumferential mucosal necrosis with congested and edematous mucosa (Fig. 3). The results of the postoperative examinations are shown in Table 1. There was no sign of congenital hematologic disease (protein C/S deficiency, antithrombin III deficiency), autoimmune disease (antiphospholipid antibody syndrome), or other complications such as collagen disease or vasculitis. Postoperatively, the patient’s general condition was stable and she began rehabilitation on the second postoperative day; however, she became aware of respiratory distress and developed cardiopulmonary arrest. Cardiopulmonary resuscitation was performed and spontaneous circulation was restored. Echocardiography showed compression of the left ventricle by the right ventricle, and PE was strongly suspected. The circulatory system was extremely unstable, and veno-arterial extracorporeal membrane oxygenation was initiated via the right femoral artery and vein. Subsequent contrast angiography of the right and left pulmonary arteries via the right internal jugular vein revealed bilateral thrombus transillumination images (Fig. 4). Based on these findings, a diagnosis of PE was made. In addition, lower extremity echocardiography showed deep vein thrombosis formation, mainly in the left popliteal vein. Thrombolysis was relatively contraindicated in the postoperative period, and thrombus aspiration was therefore performed as much as possible, followed by anticoagulation with heparin and switching to edoxaban on day 11 after clot retrieval. The patient subsequently recovered with no neurological damage and was discharged on day 26 after the procedure. A summary of the course of treatment after hospitalization is shown in Fig. 5. The patient received lower extremity echocardiography at 3 months and computed tomography scan at 5 months after discharge, and resolution of the DVT/PE and no exacerbation of PVT were confirmed.

**Table 1:** List of blood test results in perioperative period

		Standard values	
Related to hematological disease			
Protein C	97%	(70–150)	
Protein S	59%	(59–143)	
Antithrombin-III	84%	(81–123)	
Total homocysteine	8.7 nmol/ml	(5.3–15.2)	
Related to collagen disease			
Anti-cardiolipin antibody	< 4.0 U/ml	(0–12.3)	
C-ANCA	< 0.6 U/ml	(0–3.4)	
P-ANCA	< 0.2 IU/ml	(0–3.4)	
Cryoglobulin qualitative	–		
Rheumatoid factor	< 5 IU/ml	(0–15)	
Anti-CCP antibody	< 0.5 U/ml	(0–4.4)	
Anti-dsDNA antibody	< 1.2 IU/ml	(0–12)	
Anti-RNP antibody	< 2.0 U/ml	(0–9.9)	
Anti-Sm antibody	< 1.0 U/ml	(0–9.9)	
Anti-SS-A antibody	< 1.0 U/ml	(0–9.9)	
Antinuclear antibodies	–		

ANCA, anti-neutrophil cytoplasmic antibody; CCP, cyclic citrullinated peptide; RNP, ribonucleoprotein

## Discussion

PE and deep vein thrombosis are broadly classified as VTEs. In terms of pathophysiology, these thromboembolisms are caused by the interrelation of Virchow’s triad of blood hypercoagulability of blood, altered blood flow in the vessels, and vessel wall injury/endothelial damage [6]. Thromboembolism is approved with alone or in combination with these signs.

The risk factors for PVT include cancer (gastrointestinal, hepatobiliary, or pancreatic cancer), chronic liver disease (especially liver cirrhosis with portal hypertension), and non-malignant, non-cirrhotic disease (e.g., protein C/S deficiency) [7–9]; however, idiopathic PVT with no obvious cause may also occur and has been reported to account for about 14%–25% of all PVT cases [8, 10]. The current patient had no obvious cancer, liver disease, or other thrombogenic factors that might have been considered as a risk factor for PVT, and she was therefore diagnosed with idiopathic PVT.

PVT is generally classified as acute (symptom duration < 60 days, absence of portal cavernoma, and portal hypertension) or chronic (symptom duration > 60 days, evidence of portal cavernoma, or complications of portal hypertension) [2, 11]. Intestinal necrosis is a symptom of acute PVT [11, 12], and the location of the thrombus is related to the development of intestinal necrosis. Acosta et al. reported that thrombus expansion into the mesenteric vein was closely related to the development of intestinal necrosis [13]. In the present case, the thrombus was thought to have developed into MVT, which converted to acute PVT and caused intestinal necrosis; however, a portal cavernoma was present, which indicated chronic PVT.

The prognosis of PVT is relatively good in patients without cancer or cirrhosis, with a 1-year mortality rate of only 8%, compared with 26% in patients with cancer or cirrhosis [1]. Notably however, the mortality rate of acute PVT with intestinal ischemia is as high as 20–50% [14, 15]. The current patient suffered from intestinal necrosis, followed by postoperative PE leading to cardiac arrest, suggesting that acute PVT with intestinal necrosis may have a poor prognosis.

Ogren et al. reported that PVT was associated with a 2.9-fold increased risk of VTE (95% confidence interval 2.2–3.7, *P* < 0.001) [16]. Compared with cirrhosis or cancer as known risk factors for PVT, idiopathic PVT showed the strongest association with concomitant VTE (odds ratio 3.3, 95% confidence interval 1.3–7.9, *P* = 0.009). The mortality rate of VTE is also high. The incidence of fatal cardiac arrest after PE rises to 70% within the first hour of onset, with an overall mortality rate up to 95% [17, 18]. Despite this high mortality rate, it has been reported that the mortality rate of untreated PE is about 30%–35%, which can be improved to 1–20% with appropriate intervention [4, 5]. In general, when a patient with cardiac arrest is unresponsive to oxygen and pharmacologic therapy, a percutaneous cardiopulmonary support device should be introduced promptly to stabilize respiratory and circulatory failure [19]. In the presence of shock or cardiac arrest, thrombolytic therapy may be considered in addition to anticoagulation, while surgical or catheter-based thrombectomy may also be an option in patients with a bleeding risk [6]. The current patient had undergone partial resection of the small intestine and was thus at risk of bleeding, and catheter thrombectomy in addition to anticoagulation was performed, with a good outcome. In cases of idiopathic PVT, it is important to search for complications of VTE, given that an early diagnosis and prompt introduction of appropriate intervention may improve the patient’s prognosis.

Long-term control of PVT requires anticoagulant therapy and regular monitoring to prevent further thrombus formation. Antithrombin III was first covered by insurance for the treatment of PVT in Japan in 2017. In recent years, the use of direct oral anticoagulants has also been reported, with comparable outcomes to heparin and warfarin [20]. Permanent anticoagulant therapy has been recommended in patients with a history of intestinal ischemia [21]. There are currently no established follow-up criteria for PVT, but patients may receive an initial follow-up imaging study within 3–6 months after the start of treatment to assess the response to anticoagulation therapy and the status of the thrombus [22]. The present patient accordingly underwent echocardiography and computed tomography examinations within 6 months after discharge. Doctors may also consider prescribing a direct oral anticoagulant permanently, unless there are specific side effects such as bleeding. If there is no significant change in the thrombus, a longer interval between examinations would be considered. Appropriate perioperative VTE prophylaxis is required in patients with a history of idiopathic PVT, as in this case. Patients undergoing major surgery (including all abdominal surgery) with a history of VTE or a predisposition to thrombosis are usually at highest risk of DVT, which can be prevented by early postoperative weaning combined with the use of elastic stockings or intermittent pneumatic compression and anticoagulation therapy [23]. In the current case, we did not initiate preoperative anticoagulation because the patient required emergency surgery and the risk of bleeding was considered high; however, in routine surgery, it would be desirable to take preventive measures with preoperative anticoagulant therapy, even if the PVT was asymptomatic.

## Conclusions

We experienced a case of idiopathic PVT complicated by VTE including PE, which is a potentially fatal condition. Although this patient was fortunately saved by prompt intervention, it is advisable to search for complications of VTE as soon as possible after the diagnosis of idiopathic PVT.

## Data Availability

The data set supporting this article is available upon reasonable request from the corresponding authors.

## Abbreviations

CPR: Cardiopulmonary resuscitation
MVT: Mesenteric vein thrombosis
PE: Pulmonary embolism
PVT: Portal vein thrombosis
VTE: Venous thromboembolism
